# Supplementary material for: Two Laminaria japonica Fermentation Broths Alleviate Oxidative Stress and Inflammatory Response Caused by UVB Damage: Photoprotective and Reparative Effects
Source: Mar Drugs. 2022 Oct 20;20(10):650. doi: 10.3390/md20100650 (PMC9605345; doi:10.3390/md20100650)
Supplement: Supplementary file 1 [file marinedrugs-20-00650-s001.zip › marinedrugs-1968606-supplementary.pdf]

The degree of UVB damage to HaCaT cells

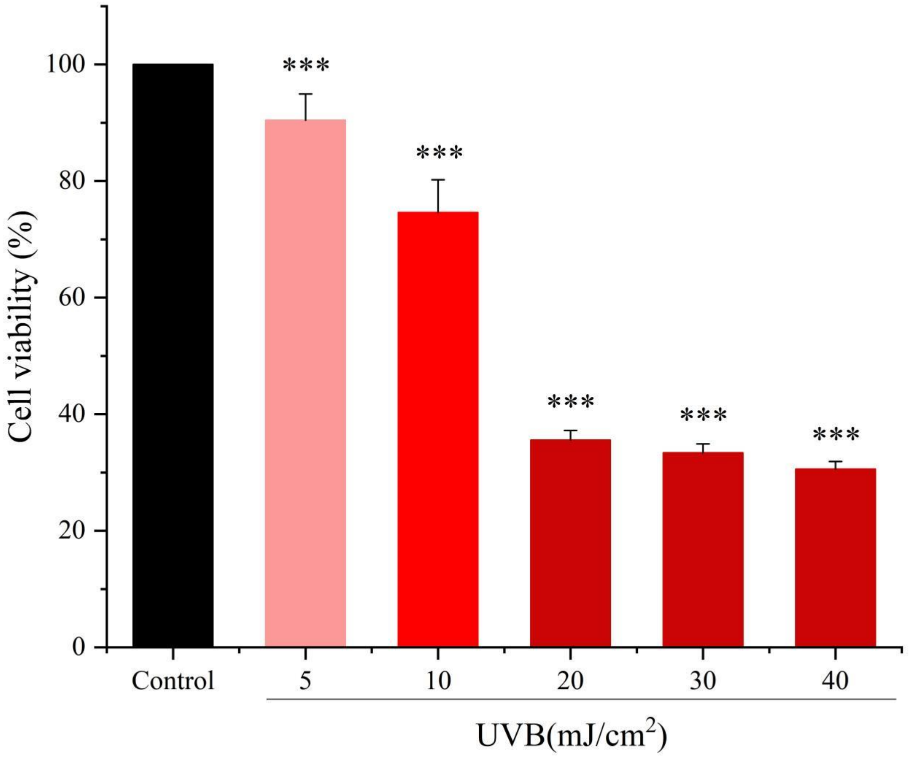

Figure S1. Effects of different doses of UVB on the survival rate of HaCaT cells. ***$p<0.001$, significantly different compared with the control group.

UVB is the main ultraviolet wavelength band that causes inflammatory damage to cells and skin. It can reach the epidermis of human skin and cause oxidative damage or even death of epidermal cells. It is a typical type of photodamage. Figure S1 shows the survival rate of HaCaT cells after different doses of UVB (5-40 mJ/cm^2). As shown in the Figure 3, HaCaT cell viability was negatively correlated with UVB dose. The cell viability was 90.42% and 74.59% when UVB energy was 5 and 10 mJ/cm^2, respectively. Afterwards, with the increase of UVB dose, the cell viability was lower than 50%. Based on the above data, we choose 10 mJ/cm^2 UVB damage dose for subsequent experiments.
